# Salinity altered root distribution and increased diversity of bacterial communities in the rhizosphere soil of Jerusalem artichoke

**DOI:** 10.1038/srep20687

**Published:** 2016-02-08

**Authors:** Hui Yang, Jinxiang Hu, Xiaohua Long, Zhaopu Liu, Zed Rengel

**Affiliations:** 1 Jiangsu Provincial Key Laboratory of Marine Biology, College of Resources and Environmental Sciences, Nanjing Agricultural University, Nanjing 210095, P.R. China; 2 Soil Science and Plant Nutrition, School of Earth and Environment, The University of Western Australia, 35 Stirling Highway, Crawley WA 6009, Australia

## Abstract

The interaction between roots and bacterial communities in halophytic species is poorly understood. Here, we used Jerusalem artichoke cultivar Nanyu 1 (NY-1) to characterise root distribution patterns and determine diversity and abundance of bacteria in the rhizosphere soil under variable salinity. Root growth was not inhibited within the salinity range 1.2 to 1.9 g salt/kg, but roots were mainly confined to 0–20 cm soil layer vertically and 0–30 cm horizontally from the plant centre. Root concentrations of K^+^, Na^+^, Mg^2+^ and particularly Ca^2+^ were relatively high under salinity stress. High salinity stress decreased soil invertase and catalase activity. Using a next-generation, Illumina-based sequencing approach, we determined higher diversity of bacteria in the rhizosphere soil at high than low salinity. More than 15,500 valid reads were obtained, and *Proteobacteria, Acidobacteria, Bacteroidetes* and *Actinobacteria* predominated in all samples, accounting for >80% of the reads. On a genus level, 636 genera were common to the low and high salinity treatments at 0–5 cm and 5–10 cm depth. The abundance of *Steroidobacter* and *Sphingomonas* was significantly decreased by increasing salinity. Higher Shannon and Chao 1 indices with increasing severity of salt stress indicated that high salt stress increased diversity in the bacterial communities.

Soil salinity is one of the most important abiotic stresses limiting the growth and crop production worldwide^1^. It was estimated that about 20% (45 million ha) of irrigated land, producing 1/3 of the world food production, is salt affected^2^. In the past few decades, soil salinization reduced the world’s production of major crops by more than 50%. By 2050, it is estimated that 50% of the world’s arable land will be affected by salinity^3^,^4^. Thus, understanding salt-tolerance mechanisms and developing salt-tolerant crops are essential for maintaining the world’s food security^5^.

The root is an important plant organ in direct contact with the soil solution, thus being the first to encounter the saline medium^1^. The root distribution pattern in soil is reflection of the plant ecological adaptation, and may increase a chance of plant survival under stress^6^. However, root responses to soil salinity in halophytes and their relation with plant growth are poorly understood.

Jerusalem artichoke (*Helianthus tuberosus* L.) belongs to sunflower family; it is a perennial tuberous plant rich in inulin and a potential energy crop^7^. Distributed throughout the world, Jerusalem artichoke has showed wide ecological adaptability. It has a number of advantageous characteristics over traditional agricultural crops, including high growth rate, good tolerance to frost, drought and poor-fertility soil, strong resistance to pests and diseases, and the minimal-to-zero fertilizer requirements. Unlike grain crops, Jerusalem artichoke can grow well in barren, poor-quality land^8^. Jerusalem artichoke is a versatile plant, potentially achieving a high biomass yield of tubers and stalks^9^. The plant produces underground tubers that are rich in inulin^10^ (amounting to 10–20% of fresh tuber weight).

Plant growth is affected by soil enzyme activity and soil microorganisms. Enzymes are secreted by microorganisms, plants and soil animals, and they regulate many soil biological processes^11^. Soil microorganisms are recognized as a key factor influencing plant growth, but it is challenging to fully characterize soil microbial communities. In particular, there is little knowledge about the structure of microbial communities in the rhizosphere of halophyte plants growing in salt-affected soils.

In this paper, we characterized Jerusalem artichoke root distribution patterns and bacterial communities in the rhizosphere under different salinity stress conditions to shed light on the response mechanisms induced by salt stress in Jerusalem artichoke roots.

## Methods

### Plant material and growth conditions

Jerusalem artichoke (*Helianthus tuberosus* L.) was collected from Experimental Station “863” at Dafeng, 4 km from the Yellow Sea shore in Jiangsu Province of China (32°59′N, 120°49′E). The area is located in the subtropical monsoon climate zone; the annual precipitation is 1,058 mm, mainly concentrated in the June-to-August rainy season. Four fields with different salinity were selected (in g salt/kg soil: S1 = 1.2 ~ 1.9; S2 = 1.6 ~ 1.8; S3 = 2.1 ~ 2.6; S4 = 2.6 ~ 3.0), and each field included three replicate plots. As annual, Jerusalem artichoke was planted in March and sampled in August 2014. Jerusalem artichoke cultivar was Nanyu 1 (NY-1), and its growth period was about 230 d. Plant spacing was 60 cm inter-row, and the intra-row distance between plants was 50 cm.

### Root sampling and analyses

Three uniform plants were selected in each salinity plot. Root samples were collected by soil layer (0–5, 5–10, 10–15 and 15–20 cm from the surface) using a sampling tube, and in different sections horizontally (0–10, 10–20 and 20–30 cm from the plant center in each layer). Samples were sealed in plastic bags and transported to the laboratory, where visible roots were collected from soil manually, followed by applying water to collect fine roots on nylon gauze (0.8-mm mesh). Root samples were stored in zip-lock bags and marked for further investigations. A detailed description of the root collection procedures was given in the previous reports^12^,^13^.

Roots in each soil layer and horizontal section were divided into three size classes based on root diameter measured using a vernier caliper (0.02-mm resolution): fine roots (<2 mm), medium roots (2–4 mm) and coarse roots (>4 mm). The root length density (RLD, root length per unit volume of soil, m·m^−3^) of each layer was determined as follows: RLD_ni_ = L_ni_/V_ni_, where L was the root length in each soil block, and V_ni_ (Vni = π(r^2^_ni_-r^2^_n(i−1)_)) was the volume of soil, “n” was horizontal section (n = 1,2,3), and “i” was the vertical stratification (i = 1,2,3,4). Root samples were oven-dried at 80 °C for at least 48 h and weighed. Concentrations of Ca^2+^, K^+^, Na^+^ and Mg^2+^ in roots were determined by Inductively Coupled Plasma Atomic Emission Spectrometer (ICP-AES, Optima 2100DV, Pekin-Elmer, USA) after digesting^14^.

### Soil sampling and analyses

Soil samples were collected from each soil layer (0–5, 5–10, 10–15 and 15–20 cm from the surface) using a cylindrical soil core. The soil samples were kept in zip-lock bags for transport to the laboratory. Soil water content was measured using a subsample of approximately 10 g before and after drying at 80 °C for at least 48 h. Remaining soil samples were sieved through a 1-mm sieve and air-dried at least one week for the following analyses. Soil pH and soluble salt content were measured using a 1:5 (soil: water) suspension. The total Ca^2+^, K^+^, Na^+^ and Mg^2+^ concentrations in the same supernatant were determined by ICP-AES^15^.

### Tuber growth and biomass

In mid-December 2014, three tuber samples were collected from each of the four saline treatments in the same layers (vertically) and sections (horizontally) as for the root samples, then placed in plastics bags and transported to the laboratory. Tuber samples were oven-dried at 80 °C for at least 96 h and weighed.

### Soil enzyme activity

Soil urease activity was detected using improved sodium phenate and sodium hypochlorite colorimetry^16^. Invertase activity was determined by the method that involved the colorimetric determination of reducing sugars that react with 3,5-dinitrosalicylic acid upon incubation of soil in buffered (0.17 M modified universal buffer, pH 5.5) sucrose solution and toluene at 37 °C for 24 h^17^. Catalase activity was based on the recovery rates of H_2_O_2_, and the residual H_2_O_2_ was determined by titration with KMnO_4_ in the presence of H_2_SO_4_^18^,^19^.

### Soil bacterial communities

#### Soil sampling

According to Riley and Barber^20^,^21^, whole plants were extracted from fields. The bulk soil was obtained by gently shaking roots. The rhizosphere soil was then collected as soil that adhered to roots. The rhizosphere soil samples were collected from two salinity treatments (S1 and S4) at 0–5 cm and 5–10 cm depth. The rhizosphere soil samples were transferred into DNA-free polythene bags, kept on dry ice for transport to the laboratory, and were then stored at −20 °C for biological and biochemical analyses. Bulk soil samples were kept in zip-lock bags, transferred to the laboratory, and air-dried at room temperature for biological and biochemical analyses^22^.

#### Soil DNA extraction

Three replicate samples were randomly picked from each treatment in the lab and used for DNA extraction. Soil DNA was extracted from 0.25 g of soil (after passing a 1-mm sieve) using a PowerSoil DNA Isolation Kit (MO BIO Laboratories, Inc., Carlsbad, CA, USA), according to the manufacturer’s instructions. The extracted soil DNA was dissolved in 100 mL TE buffer (Tris-hydrochloride buffer, pH 8.0, containing 1.0 mM EDTA), quantified by ND1000 and stored at −80 °C before using^23^.

#### Bacterial 16S rRNA gene amplification and Illumina Sequencing

Primers 577F (5′-AYTGGGYDTAAAGNG-3′) and 926R (5′-CCGTCAATTCMTTTRAGT-3′) targeting the regions (V3-V4) of the 16S rRNA gene were used for PCR, because sequences in that regions provided the greatest diversity at the domain and bacterial phylum levels^24^. Amplification reactions were performed in 25-μL volume containing 12.5 μL Premix Ex TaqTM Hot Start Version (Takara Biotechnology Co. Ltd, Dalian, China.), 0.1 μM of each primer, and 20 ng of template. Amplification was initiated at 98 °C for 3 s, followed by 35 cycles of denaturation at 98 °C for 10 s, primer annealing at 54 °C for 30 s, extension at 72 °C for 45 s, and final extension for 10 min. Amplicon pyrosequencing was performed on an Illumina MiSeq platform at LC-Bio Technology Co., Ltd, Hangzhou, Zhejiang, China. The complete data sets were deposited in the NCBI, and the GenBank accession numbers are KT783673 - KT784803.

Pairs of reads were merged from the original DNA fragments by using FLASH (version1.2.8)^25^ that was designed to merge pairs of reads when the original DNA fragments were shorter than two times the reads length. Sequencing reads were distributed to each sample according to its unique barcode. QIIME (version 1.7.0)^26^ software package (Quantitative Insights Into Microbial Ecology) and the CD-HIT pipeline were used to analyze sequences. The reads were filtered by QIIME quality filters at first. Default settings for Illumina processing in QIIME was used (r = 3 p = 0.75 total read length; q = 3; n = 0).

(p) min_per_read_length: minimum number of consecutive high-qualitybase calls to retain read (as percentage of totalread length).

(r) max_bad_run_length: maximum number of consecutive low-quality base calls allowed before truncating a read.

(n) sequence_max_n: maximum number of ambiguous (N) characters allowed in a sequence.

(q) phred_quality_score: last quality score considered low quality.

The CD-HIT pipeline was used for picking operational taxonomic units (OTUs). Sequences were assigned to OTUs at 97% similarity. Representative sequences were chosen for each OTU and taxonomic data were then assigned to each representative sequence using the RDP (Ribosomal Database Project) classifier^27^. In order to estimate Alpha Diversity, the OTU table was rarified and four metrics were calculated: Chao1 metric to estimate the richness, the Observed OTUs metric as the count of unique OTUs found in the sample, Shannon index and Simpson index^28^,^29^.

#### Data analysis

All measurements were replicated thrice as mentioned in each section. The mean values of all parameters were taken from three replicates, and the standard error of the means was calculated. For statistical analyses, one-way or two-way ANOVA, Duncan post-hoc tests (p = 0.05) and Spearman’s rank correlations were used separately for each soil layer by SPSS Statistics 19.0 (IBM, Armonk, New York, USA). The community richness index, community diversity index, data preprocessing, operational taxonomic unit-based analysis and hypothesis tests were performed using QIIME 1.7.0. The histograms were created using Microsoft Excel 2010 and all tables were made with Microsoft Word 2010 (Microsoft, Redmond, Washington, USA). Individual means were compared using the least significant difference test α = 0.05 significance level.

## Results

### Root distribution

**Figure 1:**
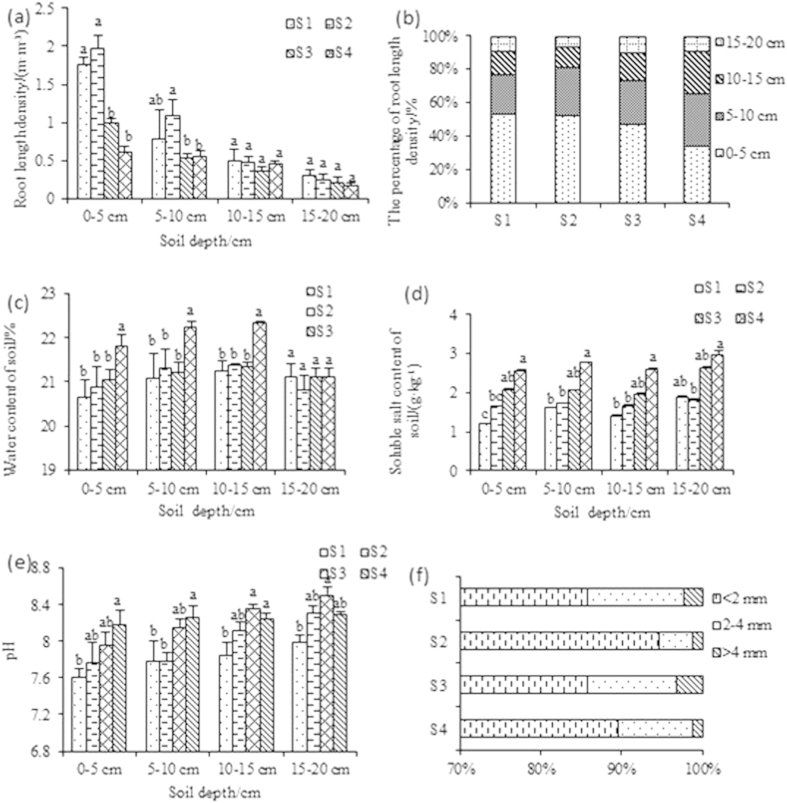
Jerusalem artichoke (cv. NY-1) root length density distribution (**a**), percentage of root length density down the soil profile (**b**), and the changes in water content (**c**), soluble salt content (**d**), and pH (**e**) at different soil depths. Also shown is the percentage of total root length in different diameter class (**f**) of Jerusalem artichoke. Data are means + SE (n  = 3). For graphs a-f, one-way ANOVA (main factor  =  salinity) followed by Duncan test (p = 0.05) was done for each soil layer separately.

The Jerusalem artichoke (NY-1) roots were mainly distributed in the top soil layer (Fig. 1a), with less than 8.5% of total root length density being in the 15–20 cm soil layer.

The root length density in the vertical direction tended to decrease with the increased salt stress as well as with soil depth (Fig. 1a). However, the root length density in the 0–5 and 5–10 cm soil layers was not significantly different between S1 and S2, and there were no significant differences among the treatments between 10–15 and 15–20 cm soil layers (Fig. 1a).

The percentage of root length density in the 0–5 cm depth layer did not significantly differ among the salinity treatments, but there was a decreasing trend with an increase in severity of salt stress (Fig. 1b). The proportion of root length density was higher in the 0–5 cm depth layer compared with the other three layers in the treatments S1, S2 and S3, but in S4 there was no significant difference between 0–5 and 5–10 cm depth (Fig. 1b).

Horizontally, NY-1 root growth reached at least 30 cm from the plant center (Table 1), with H1, H2 and H3 representing the sections 0–10, 10–20 and 20–30 cm from the plant center, respectively. In H1 section, S2H1 had highest root length density; there was no significant difference compared with S1H1, but significant differences were found in comparison with S3H1 and S4H1. A similar trend was found in H2 and H3, which meant that root growth was not affected by the increased salt stress in S2.

### Root distribution with gradients in soil moisture, salinity and pH

The soil moisture content increased with depth and reached maximum in the 10–15 cm layer, up to 23% (w/w) in the 10–15 cm of the treatment S3 (Fig. 1c). In the 0–20 cm soil layers in the S1 to S3 treatments, there were no significant differences in water moisture content, but water content was higher in the 0–15 cm soil layers in S4 than in the other three treatments (Fig. 1c). In contrast, soil soluble salt content tended to increase with soil depth, and was predictably higher with increasing severity of salt stress in the four salinity treatments (Fig. 1d). The soil pH increased with depth and with increasing soil salinity content (Fig. 1e).

Root length density had no significant correlation with soil water content (data not shown). Root length density (P = 0.005) had a significant negative correlation with soil soluble salt content. Root length density (P = 0.000) had a significant negative correlation with soil pH (data not shown).

### Root diameter classes

Jerusalem artichoke fine roots (<2 mm) accounted for the largest proportion of the total root length, followed by medium (2–4 mm) and coarse (>4 mm) roots (Fig. 1f). Fine roots had a very significant positive correlation with medium roots and coarse roots (P = 0.000), and medium roots and coarse roots were also significantly positively correlated (P = 0.000) (data not shown).

### Ion concentration in roots and soil

The concentrations of Ca^2+^ and Mg^2+^ in soil were obviously higher than those in roots. The K^+^ concentrations in soil and roots were similar, but Na^+^ concentration was lower in soil than roots (see Supplementary Fig. S1). The Na^+^ concentration was only about 3.6 mg/g in soil, and was about 3–4 times higher in roots. This result indicated that NY-1 roots can accumulate Na^+^, but not K^+^, Ca^2+^ and Mg^2+^ (see Supplementary Fig. S1).

### Tuber biomass and distribution

**Figure 2:**
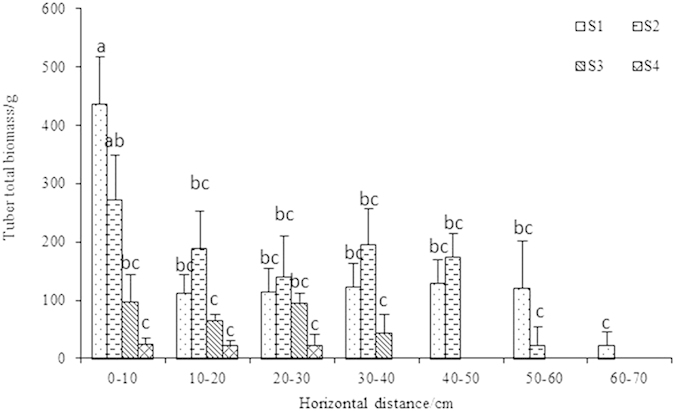
The distribution of Jerusalem artichoke (NY-1) tubers in the 0–5 cm soil layer. Means ± standard error (n = 3); means followed by different letters (one-way ANOVA followed by Duncan test, main factor = salinity) are significantly different at P ≤ 0.05.

Tubers could be found at 70-cm horizontal distance from the plant center in the 0–5 cm soil layer (Fig. 2). Tuber distribution horizontally decreased with increasing salinity (Fig. 2). Tuber biomass showed no significant difference between S1 and S2 or S3 and S4, but significant differences were observed in S1 and S2 compared with S3 and S4 (Table 2). Tuber water content at the highest salinity treatment (S4) was significantly lower than in the other treatments (Table 2).

### Soil enzyme activity

Soil urease activity gradually decreased with increasing soil depth, but catalase and invertase activities did not show such a trend (data not shown). Urease activity and soil soluble salt content had no significant correlation (Table 3). Invertase (P = 0.006) or catalase activity (P = 0.002) was negatively correlated with soil soluble salt content. Urease (P = 0.049), invertase (P = 0.027) or catalase activity (P = 0.048) was negatively correlated with soil pH. Root length density had a very significant correlation with urease (P = 0.004) and catalase activity (P = 0.003).

### Soil microbial abundance

#### Richness

**Figure 3:**
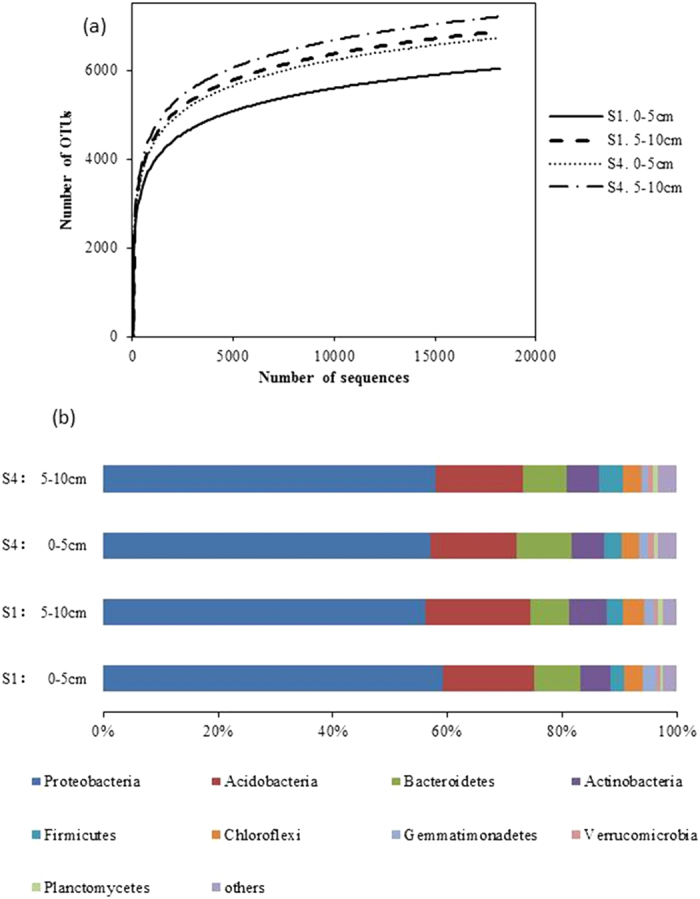
(**a**) Rarefaction curves showing the observed OTU richness (at 97% identity) of the 16S rRNA gene with increasing sequencing depth. Mean values (n = 3) were shown for the two salinity treatments (S1 and S4) and two soil depths. (**b**) Comparison of the bacterial communities at the phylum level. Relative read abundance of different bacterial phyla in bacterial communities. Sequences that could not be classified into any known group were labeled “others”.

Through a sequence optimization process, more than 15,500 valid reads were obtained for each replicate; after quality filtering, median sequence length of each read was 100 bp. In the 0–5 cm and 5–10 cm depth layers in the S4 treatment, more than 1600 additional OTUs were observed compared with the S1 treatment (Fig. 3a).

The richness indices of the bacterial communities were computed (Table 4). Higher Shannon and Chao 1 indices in S4 than S1 indicated that high severity of salt stress increased diversity in the bacterial communities (Table 4). The Simpson index is reverse of diversity (the lower the index, the greater biodiversity). Hence, lower Simpson index in S4 than S1 (Table 4) would indicate greater diversity, thus confirming the results derived from the Shannon and Chao 1 indices.

#### Taxonomic coverage

All of the sequences were classified into 32 phyla or groups by RDP (Ribosomal Database Project: http://rdp.cme.msu.edu/index.jsp). The overall bacterial composition of different samples was similar, but the distribution of each phylum in group varied (Fig. 3b). In all samples, *Proteobacteria, Acidobacteria, Bacteroidetes*, and *Actinobacteria* were the four most dominant phyla, accounting for >80% of the reads. Compared with the high salt treatment (S4) averaged over two depths, the low salt treatment (S1) had a significantly higher percentage of *Proteobacteria* (1.3-fold), *Acidobacteria* (1.4-fold), *Chloroflexi* (1.4-fold) and *Gemmatimonadetes* (1.9-fold), and a lower percentage of *Firmicutes* (1.1-fold) and *Verrucomicrobia* (1.2-fold). The percentages of *Bacteroidetes, Actinobacteria* and *Planctomycetes* were similar in S1 and S4.

On a genus level, all 643 detected genera were found in all the samples, except for *Loktanella, Salinimicrobium, Kordiimonas*, and *Muricauda* that were not detected in S1, and *Aquabacterium, Gp13* and *Klebsiella* that were not detected in S4.

The 72 genera showing significant differences among the samples were listed in Table 5. *Steroidobacter, Sphingomonas, Kofleria, Pseudolabrys, Desertibacter, Gaiella, Dongia, Iamia, Flavobacterium, Tistlia, Janthinobacterium, Blastobacter, Aminobacter* and *Pseudoxanthomonas* were significantly higher in S1 than S4. In contrast, *Pelagibius, Rhodoligotrophos, Thiohalomonas, Limimonas, Thermoleophilum, Roseicyclus, Azoarcus, Euzebya, Fulvivirga, Haliea, Rubribacterium* and *Thioalkalispira* were higher in S4 than S1.

## Discussion

Jerusalem artichoke has strong salt tolerance that is largely related to the ecological and biological characteristics of the root system^30^. The root architecture is important for plants to access soil resources, and morphological and physiological adaptation of the root system under stress conditions may result in continuation of nutrient absorption and utilization^31^. Roots of Jerusalem artichoke (NY-1) were mainly distributed in the 0–15 cm depth layer (Fig. 1a), indicating root growth in the upper soil layers to potentially avoid salinity stress. Indeed, compared with shallow layers, the 15–20 cm depth layer had higher salinity, lower water content and higher pH in each salt treatment, resulting in poor root growth in that layer.

In the present study under high salt stress (S4), roots extended horizontally more than into depth (Table 1, Fig. 1a), suggesting that NY-1 escaped from salt stress by preferentially elongating roots horizontally rather than going deep. Root extension in horizontal direction was beyond 30 cm, suggesting that horizontal expansion in the absence of deep root growth might have been caused by the need for resource acquisition in a stress environment.

Soil salinity increased from the treatment S1 to S4. Root growth in the 0–10 cm soil depth layer was significantly inhibited in S3 and S4 (Fig. 1a). In addition to salinity, there might have been other factors affecting the root system distribution, such as soil fertility^32^.

Concentrations of K^+^, Ca^2+^, Na^+^ and Mg^2+^ in roots were relatively high, but did not result in root death. One potential reason is that Ca^2+^ concentration was higher than that of other ions, potentially reducing the damage^33^. So, one of the salt resistance mechanisms of Jerusalem artichoke NY-1 may be maintenance of relatively high Ca^2+^ concentration in roots (see Supplementary Fig. S1).

Tubers were distributed only in the surface soil, and were found up to 70 cm horizontally away from the plant center. Tuber distribution became restricted with increasing salinity, suggesting that roots extending in saline soils may the capacity to grow tubers impaired with increasing salinity stress.

Soil enzymes are involved in biological cycling and soil fertility, so they are crucial indicators of soil biochemistry^34^. With the soil soluble salt content increasing, invertase and catalase activities were both reduced significantly (Table 3), suggesting impaired ecosystem functions. Catalase is present in almost all aerobic microorganisms^35^,^36^. Because of a decrease in abundance of certain groups of microbes at high salinity (Tables 4 and 5), the activity of soil catalase might have decreased. For example, *Sphingomonas* is obligate aerobic and produces redox mediators^37^. Some species of genus *Steroidobacter* were strictly aerobic, and had a positive relationship with soil catalase activity^38^.

The 16S rRNA gene sequencing results indicated a positive relationship between increasing salinity and enhancing biodiversity. This might be attributed to proliferation of halophylic bacteria in soil. Higher percentages of *Proteobacteria, Acidobacteria, Chloroflexi* and *Gemmatimonadetes* and lower percentages of *Firmicutes* and *Verrucomicrobia* were observed in low-salt than high-salt soil. The phylum *Verrucomicrobia* is widespread, but a poorly characterized group of bacteria that occur in a wide range of habitats including soils, aquatic systems, marine sediments, and hot springs; some even occur as endosymbionts^39^. In our study, the *Verrucomicrobia* abundance decreased from 1.11% to 0.64% with the salinity increasing from the treatment S1 to S4. More research is required to determine whether this phylum has specific roles in the rhizosphere soil.

In this study, *Loktanella* and *Kordiimonas* were only found at high soil salinity. *Loktanella* is a genus of the Rhodobacteraceae that have been reported to be halophilic and found in seawater^40^. *Kordiimonas* was isolated from the marine environment and could survive in oligotrophic environment^41^.

In this study, *Lysobacter* was among dominant populations in the Jerusalem artichoke (NY-1) rhizosphere soil. In other studies, *Lysobacter* was found in soil and water and had biolytic activity against a variety of pathogenic fungi, bacteria, and nematodes^42^. *Lysobacter* not only colonized the rhizosphere of various plants^43^, but also secreted a variety of antibiotics^44^,^45^, exocellular enzymes and biologically-active material^46^,^47^ to inhibit bacterial growth, thus controlling plant diseases^48^. Unfortunately, the understanding related to rhizosphere populations of *Lysobacter* is still in its infancy.

*Sphingomonas* belongs to a group of Gram-negative, rod-shaped, chemoheterotrophic, strictly aerobic bacteria that are widely distributed in nature, having been isolated from many different land and water habitats, as well as from plant root systems, clinical specimens, and other sources; they have the capacity to survive at low nutrient concentrations, as well as to metabolize a wide variety of carbon sources^49^. *Sphingomonas* was one of the most effective microbial groups to clean up the toxic substances in soil^50^. Some *Sphingomonas* strains showed characteristics of nitrogen fixation and denitrification, suggesting they played an important role in the nitrogen cycle^49^. In the present study, greater abundance of *Sphingomonas* was found at low compared with high soil salinity, indicating their potential low salt tolerance.

## Additional Information

**How to cite this article**: Yang, H. *et al.* Salinity altered root distribution and increased diversity of bacterial communities in the rhizosphere soil of Jerusalem artichoke. *Sci. Rep.* **6**, 20687; doi: 10.1038/srep20687 (2016).

## Supplementary Material

Supplementary Information

## Figures and Tables

**Table 1:** The horizontal distribution of root length density of Jerusalem artichoke (NY-1) with salinity increasing from S1 to S4 (H1, H2 and H3 represented the sections 0–10, 10–20 and 20–30 cm from the plant center respectively).

**Sections**	**Root length density/(m·m**^**−3**^)	**P**_**0.05**_
S2H1	2.10 ± 0.14	a
S1H1	1.91 ± 0.04	a
S3H1	1.19 ± 0.21	b
S2H2	0.94 ± 0.23	bc
S1H2	0.78 ± 0.14	cd
S4H1	0.77 ± 0.06	cd
S2H3	0.74 ± 0.18	cd
S1H3	0.62 ± 0.20	de
S4H2	0.52 ± 0.05	de
S4H3	0.50 ± 0.13	de
S3H2	0.43 ± 0.02	e
S3H3	0.43 ± 0.11	e

Means ± standard error (n = 3); means followed by different letters (two-way ANOVA for salinity and horizontal distance followed by Duncan test) are significantly different at P ≤ 0.05.

**Table 2:** Tuber dry weight and tuber water content of Jerusalem artichoke (NY-1) in different salinity treatments (salinity increasing from S1 to S4).

	**S1**	**S2**	**S3**	**S4**
Tuber dry weight/g	221 ± 22a	196 ± 41a	68 ± 3b	24 ± 5b
Tuber water content/%(w/w)	79 ± 2.27a	80 ± 0.82a	77 ± 1.27a	68 ± 2.83b

Means ± standard error (n = 3); means followed by different letters in a row (one-way ANOVA followed by Duncan test) are significantly different at P ≤ 0.05.

**Table 3:** Correlation coefficients between soil soluble salt content, pH or Jerusalem artichoke (NY-1) root length density with urease, invertase or catalase activity in soil.

	**Urease**	**Invertase**	**Catalase**
Soil soluble salt content	−0.18	−0.39**	−0.43**
Soil pH	−0.29*	−0.32*	−0.29*
Root length density	0.41**	0.28	0.42**

Note: *significant at 5% level, **significant at 1% level.

**Table 4:** Comparison of the estimated operational taxonomic unit (OTU) richness and diversity indices of the 16S rRNA gene libraries for clustering at 97% identity as obtained from the pyrosequencing analysis.

**Salinity treatment**	**Soil depth**	**Observed OTUs**	**Shannon index**	**Chao 1**	**Simpson’s diversity (10**^**−2**^)
S1	0–5 cm	4349^b^	12.09^c^	9462196^c^	0.023^a^
	5–10 cm	5106^b^	12.32^b^	13085704^bc^	0.020^b^
S4	0–5 cm	5993^a^	12.55^a^	18007051^b^	0.017^c^
	5–10 cm	6833^a^	12.73^a^	23645582^a^	0.015^c^

Means (n = 3). Means followed by different letters (one-way ANOVA followed by Duncan test for the salinity treatment, done for each salinity level and soil depth separately) are significantly different at P ≤ 0.05.

**Table 5:** The genera showing significant differences in percent abundance among the samples (low and high salinity and two different soil layers).

**Taxon**	**Salinity treatment S1 (1.2–1.9 g salt/kg soil)**		**Salinity treatment S4 (2.6–3.0 g salt/kg soil)**	
	**0–5 cm depth (%)**	**5–10 cm depth (%)**	**0–5 cm depth (%)**	**5–10 cm depth (%)**
Steroidobacter	2.63 ± 0.11ab	2.93 ± 0.22a	1.88 ± 0.52bc	1.54 ± 0.12c
Sphingomonas	2 .51 ± 0.61ab	3.29 ± 0.38a	1.26 ± 0.20bc	1.06 ± 0.14c
Thioprofundum	1.10 ± 0.28b	1.28 ± 0.09ab	1.57 ± 0.18ab	1.98 ± 0.24a
Pelagibius	0.93 ± 0.26b	0.91 ± 0.05b	1.73 ± 0.37ab	2.14 ± 0.27a
Blastocatella	1.54 ± 0.12ab	1.04 ± 0.01b	2.28 ± 0.58a	2.00 ± 0.26ab
Rhodoligotrophos	0.86 ± 0.14b	0.93 ± 0.08b	1.13 ± 0.23ab	1.56 ± 0.10a
Nitrosospira	1.11 ± 0.11ab	1.14 ± 0.11a	0.67 ± 0.33ab	0.49 ± 0.06b
Nitriliruptor	0.57 ± 0.13bc	0.40 ± 0.02c	0.83 ± 0.21b	1.30 ± 0.04a
Albidovulum	0.59 ± 0.12b	0.65 ± 0.07ab	0.91 ± 0.15ab	1.00 ± 0.04a
Gp3	0.77 ± 0.10b	1.03 ± 0.04a	0.66 ± 0.09b	0.60 ± 0.06b
Nitrospira	0.67 ± 0.04ab	0.88 ± 0.04a	0.61 ± 0.10b	0.78 ± 0.09ab
Aciditer	0.72 ± 0.01a	0.75 ± 0.05a	0.58 ± 0.03b	0.60 ± 0.02b
Kofleria	0.88 ± 0.09a	0.85 ± 0.03a	0.60 ± 0.05b	0.61 ± 0.08b
Thiohalomonas	0.39 ± 0.15b	0.32 ± 0.06b	0.70 ± 0.14ab	0.93 ± 0.14a
Limimonas	0.39 ± 0.12c	0.31 ± 0.01bc	0.82 ± 0.23ab	0.98 ± 0.08a
Dongia	0.65 ± 0.09b	0.92 ± 0.04a	0.35 ± 0.09c	0.31 ± 0.05c
Lewinella	0.49 ± 0.11b	0.57 ± 0.04b	0.58 ± 0.09b	0.91 ± 0.05a
Salisaeta	0.44 ± 0.06b	0.46 ± 0.03b	0.60 ± 0.08b	0.91 ± 0.05a
Pseudolabrys	0.54 ± 0.01ab	0.66 ± 0.09a	0.39 ± 0.03b	0.43 ± 0.03b
Desulfovermiculus	0.50 ± 0.02ab	0.53 ± 0.06a	0.34 ± 0.08b	0.39 ± 0.04ab
Thermoleophilum	0.31 ± 0.06b	0.29 ± 0.01b	0.45 ± 0.04a	0.44 ± 0.02a
Desertibacter	0.54 ± 0.07a	0.54 ± 0.02a	0.35 ± 0.04b	0.29 ± 0.05b
Roseicyclus	0.30 ± 0.06b	0.25 ± 0.03b	0.65 ± 0.09a	0.50 ± 0.04a
Litorilinea	0.38 ± 0.03ab	0.32 ± 0.07b	0.45 ± 0.02ab	0.47 ± 0.04a
Azoarcus	0.29 ± 0.07b	0.26 ± 0.03b	0.57 ± 0.12a	0.65 ± 0.04a
Euzebya	0.26 ± 0.06b	0.21 ± 0.01b	0.40 ± 0.09ab	0.51 ± 0.04a
Gaiella	0.42 ± 0.08a	0.42 ± 0.02a	0.24 ± 0.03b	0.22 ± 0.04b
Fulvivirga	0.24 ± 0.06bc	0.14 ± 0.01c	0.50 ± 0.18ab	0.73 ± 0.01a
Iamia	0.32 ± 0.01ab	0.39 ± 0.03a	0.25 ± 0.04b	0.24 ± 0.02b
Haliea	0.23 ± 0.05bc	0.18 ± 0.01c	0.37 ± 0.08ab	0.52 ± 0.03a
Flavobacterium	0.44 ± 0.06a	0.32 ± 0.03ab	0.13 ± 0.02c	0.23 ± 0.04bc
Pseudofulvimonas	0.34 ± 0.03ab	0.21 ± 0.03b	0.44 ± 0.05a	0.32 ± 0.03ab
Rhodoplanes	0.26 ± 0.02ab	0.32 ± 0.04a	0.21 ± 0.01b	0.23 ± 0.04ab
Rubribacterium	0.21 ± 0.07b	0.19 ± 0.04b	0.45 ± 0.16a	0.37 ± 0.05ab
Tistlia	0.28 ± 0.01ab	0.33 ± 0.05a	0.18 ± 0.05b	0.16 ± 0.03b
Janthinobacterium	0.38 ± 0.08a	0.66 ± 0.15a	0.06 ± 0.03b	0.09 ± 0.01b
Blastobacter	0.34 ± 0.09a	0.31 ± 0.24a	0.12 ± 0.02b	0.13 ± 0.09b
Pseudoxanthomonas	0.43 ± 0.11a	0.34 ± 0.05a	0.06 ± 0.02b	0.11 ± 0.00b
Porticoccus	0.14 ± 0.03b	0.10 ± 0.02b	0.21 ± 0.02b	0.35 ± 0.07a
Oceanibaculum	0.13 ± 0.04b	0.21 ± 0.03ab	0.2 ± 0.04ab	0.31 ± 0.04a
Skermanella	0.22 ± 0.02b	0.32 ± 0.03a	0.06 ± 0.01c	0.06 ± 0.01c
Levilinea	0.16 ± 0.01ab	0.11 ± 0.02b	0.17 ± 0.02ab	0.21 ± 0.04a
Filomicrobium	0.16 ± 0.01ab	0.13 ± 0.03b	0.14 ± 0.01ab	0.19 ± 0.01a
Shinella	0.22 ± 0.07ab	0.29 ± 0.02a	0.08 ± 0.01c	0.10 ± 0.03bc
Brevundimonas	0.26 ± 0.06a	0.21 ± 0.02ab	0.12 ± 0.06ab	0.08 ± 0.05b
Pimelobacter	0.19 ± 0.05a	0.17 ± 0.01ab	0.12 ± 0.03ab	0.07 ± 0.02b
Terrimonas	0.26 ± 0.06a	0.37 ± 0.04a	0.08 ± 0.03b	0.07 ± 0.02b
Gp26	0.12 ± 0.01b	0.16 ± 0.02ab	0.14 ± 0.03b	0.22 ± 0.03a
Elioraea	0.14 ± 0.01ab	0.18 ± 0.01a	0.11 ± 0.02bc	0.07 ± 0.01c
Hoeflea	0.10 ± 0.02ab	0.06 ± 0.01b	0.13 ± 0.03ab	0.18 ± 0.05a
Sphingopyxis	0.20 ± 0.04ab	0.21 ± 0.04a	0.09 ± 0.03b	0.10 ± 0.02b
Thioclava	0.10 ± 0.02ab	0.06 ± 0.00b	0.18 ± 0.04a	0.17 ± 0.04a
Limnobacter	0.19 ± 0.03a	0.14 ± 0.02ab	0.07 ± 0.04bc	0.04 ± 0.00c
Arcticibacter	0.20 ± 0.04b	0.40 ± 0.08a	0.03 ± 0.02c	0.05 ± 0.02bc
Pannonibacter	0.09 ± 0.01bc	0.07 ± 0.01c	0.17 ± 0.04a	0.16 ± 0.01ab
Labrenzia	0.05 ± 0.03b	0.02 ± 0.00b	0.15 ± 0.03a	0.21 ± 0.03a
Azohydromonas	0.21 ± 0.04a	0.15 ± 0.02ab	0.10 ± 0.02b	0.08 ± 0.01b
Microbulbifer	0.07 ± 0.02b	0.08 ± 0.02b	0.12 ± 0.01b	0.19 ± 0.03a
Parasegetibacter	0.14 ± 0.01ab	0.16 ± 0.02a	0.10 ± 0.01ab	0.08 ± 0.03b
Piscinibacter	0.14 ± 0.02a	0.15 ± 0.02a	0.08 ± 0.00b	0.08 ± 0.02b
Hyphomicrobium	0.06 ± 0.03b	0.13 ± 0.01a	0.08 ± 0.01ab	0.10 ± 0.02ab
Caldilinea	0.14 ± 0.03a	0.14 ± 0.01a	0.08 ± 0.01b	0.08 ± 0.03b
Luteolibacter	0.08 ± 0.01b	0.07 ± 0.02b	0.24 ± 0.05a	0.25 ± 0.04a
Sediminibacter	0.06 ± 0.02b	0.02 ± 0.01b	0.13 ± 0.02a	0.15 ± 0.01a
Sphingobium	0.20 ± 0.07a	0.18 ± 0.03ab	0.06 ± 0.02ab	0.05 ± 0.02b
Nocardioides	0.12 ± 0.02ab	0.15 ± 0.04a	0.06 ± 0.02b	0.05 ± 0.01b
Rhodovulum	0.05 ± 0.02b	0.06 ± 0.02b	0.13 ± 0.04ab	0.17 ± 0.00a
Massilia	0.17 ± 0.05a	0.30 ± 0.07a	0.03 ± 0.01b	0.01 ± 0.00b
Thauera	0.10 ± 0.02b	0.15 ± 0.00a	0.06 ± 0.02b	0.06 ± 0.01b
Georgfuchsia	0.13 ± 0.02a	0.11 ± 0.02a	0.06 ± 0.01b	0.06 ± 0.01b
Aminobacter	0.24 ± 0.03a	0.26 ± 0.07a	0.12 ± 0.04b	0.16 ± 0.05b
Thioalkalispira	0.13 ± 0.04b	0.14 ± 0.01b	0.25 ± 0.04ab	0.31 ± 0.04a

Means ± standard error (n = 3); means followed by different letters in a row (one-way ANOVA followed by Duncan test for salinity) are significantly different at P ≤ 0.05.
