# Supplementary material for: Salinity altered root distribution and increased diversity of bacterial communities in the rhizosphere soil of Jerusalem artichoke
Source: Sci Rep. 2016 Feb 8;6:20687. doi: 10.1038/srep20687 (PMC4745076; doi:10.1038/srep20687)
Supplement: Supplementary Information [file srep20687-s1.doc]

Salinity altered root distribution and increased diversity of bacterial communities in the rhizosphere soil of Jerusalem artichoke

Hui Yang ^a^, Jinxiang Hu ^a^, Xiaohua Long ^a^ [[1]](#footnote-2)*, Zhaopu Liu ^a^, Zed Rengel ^b^

^a^ Jiangsu Provincial Key Laboratory of Marine Biology, College of Resources and Environmental Sciences, Nanjing Agricultural University, Nanjing 210095, P.R. China

^b^ *Soil Science and Plant Nutrition, School of Earth and Environment, The University of Western Australia, 35 Stirling Highway, Crawley WA 6009, Australia*


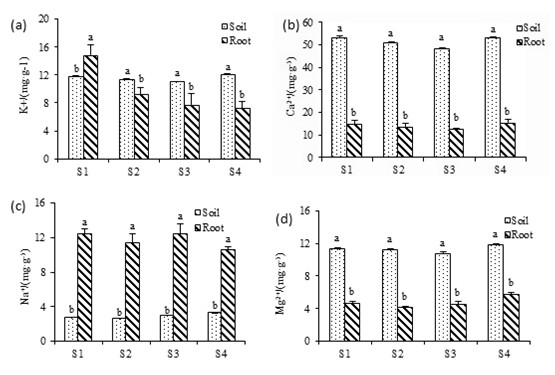


**Fig. S1**  Ion (K^+^, Ca^2+^, Na^+^ and Mg^2+^) concentration in soil and roots of Jerusalem artichoke grown at four salinity levels (S1-S4). Data are means + SE (n =3). One-way ANOVA (main factor = salinity) followed by Duncan test (p=0.05) was done.

1. ^*^Corresponding author. Email: [longxiaohua@njau.edu.cn](mailto:longxiaohua@njau.edu.cn), Tel /fax number: +86 2584396678.

   No conflict of interest in the paper. [↑](#footnote-ref-2)
